# Using an analytic auto-netnographic approach to explore the perceptions of paramedics in primary care

**DOI:** 10.29045/14784726.2024.12.9.3.21

**Published:** 2024-12-01

**Authors:** Georgette Eaton, Stephanie Tierney, Geoff Wong, Kamal R. Mahtani

**Affiliations:** Nuffield Department of Primary Care Health Sciences ORCID iD: https://orcid.org/0000-0001-9421-2845; Nuffield Department of Primary Care Health Sciences ORCID iD: https://orcid.org/0000-0002-2155-2440; Nuffield Department of Primary Care Health Sciences ORCID iD: https://orcid.org/0000-0002-5384-4157; Nuffield Department of Primary Care Health Sciences ORCID iD: https://orcid.org/0000-0002-7791-8552

**Keywords:** community of practice, paramedics, primary health care, social media

## Abstract

**Introduction:**

Paramedics in the UK are moving from emergency ambulance services into primary care, where they are employed to boost the clinical workforce. Whereas there is emerging research that seeks to understand the contribution of paramedics to the primary care workforce, there is none regarding the perceptions paramedics have regarding their role in primary care.

**Methods:**

An analytic auto-ethnography was undertaken, utilising a peripheral membership approach for online communities used by paramedics on Facebook, Reddit and Twitter (now X). Over a 3-month period (December 2021 to February 2022), the primary researcher reflected on the conversations, comments and opinions posted within these communities within a reflexive (immersion) journal, considering them against the context of her own experience.

**Results:**

Paramedics in primary care, who are generally isolated due to their geographical isolation from each other, utilise online social spaces to foster a community of practice. These forums are used to discuss their clinical role, education and experiences, as well as to consider their place within the primary care workforce.

**Conclusion:**

This is the first application of this methodology within online social spaces utilised by UK paramedics. This article also presents novel use of a peripheral membership approach within an analytic auto-netnography in public online spaces for researcher-practitioners.

## Introduction

Paramedics in the UK are traditionally associated with the provision of emergency care within an emergency ambulance service. However, changes to healthcare access for patients have resulted in a significant proportion of 999 calls involving lower-acuity presentations (NHS England, 2014), leading to a change in the role of the paramedic. As well as delivering emergency care, paramedics must also now be able to recognise and treat a range of health complaints that would typically be seen by general practitioners (GPs) in primary care. This expanded role has coincided with a move from a range of vocational training pathways to degree-level pre-registration programmes (Health and Care Professions Council, 2018) and amendments to UK legislation that support paramedics to undertake additional education in order to commence independent prescribing (NHS England, 2018).

This combination of diversifying clinical experiences in the ambulance service, as well as practitioners’ higher levels of education and ability to independently prescribe medicines, has equipped the paramedic profession to be well suited to work in primary care settings. Indeed, support for paramedics to work specifically in primary care has been outlined in key policy documents published in the past five years (NHS England, 2019a, 2019b, 2023), cementing the paramedic position in the primary care workforce. The employment of paramedics in this workforce has been in response to an increased demand for complex case management in the community (Montgomery et al., 2017). This decision is driven by challenges in recruiting and retaining GPs (Majeed, 2017), prompting workforce adaptations and creating opportunities for other clinicians to support GP roles in this setting (NHS England, 2019b; Primary Care Workforce Commission, 2015).

In order to address the gaps made evident within previous research (Eaton et al., 2020, 2021; Shannon et al., 2022), further understanding of paramedics’ perspectives of working within primary care and whether paramedics maintain their existing professional identity as they move into primary care was needed. The primary goal of this research was to understand the experiences of paramedics in UK primary care, gaining an insight into how their identity is shaped through their perceptions of their role.

## Methods

The prosaic nature of experience, and the space within which this experience is discussed, can be understood through anthropological approaches rooted in dialogues among members of the community of interest (Fricke, 2003). Netnography is a qualitative research method that seeks the same understanding in dialogues among members of social media networked communities (Kozinets, 2015).

The benefits of studying people in the social media realm are twofold. First, conversations between members of communities of interest become accessible by virtue of them taking place in online public forums (Kozinets, 2010). Second, as paramedics would typically work for different providers in geographical isolation from each other (due to the nature of primary care within the UK), social media forums serve as one of the few spaces where paramedics in this clinical setting can connect.

### Study design and procedures

This study was conducted as an independent work package within a wider realist evaluation (Eaton, 2023). From a realist epistemological perspective, knowledge is acquired through understanding a reality that exists independently of our perceptions, with empirical evidence serving as the foundation for this understanding. Employing a realist approach was essential for this study as this ontological stance acknowledges the complexity of the real-world working lives of paramedics who will (by nature of their experiences) work differently and in different contexts. A protocol was published and registered with OSF Registries (10.17605/OSF.IO/BJQXP). The study design followed the process set out by Kozinets (2019) through five procedural movements.

#### Initiation

As a paramedic working in primary care, GE opted for an approach that would enable her to examine her ‘culture from the perspective of the self’ (Adams et al., 2014, p. 46). Thus, an approach to research that ‘seeks to describe and systematically analyse (graphy) personal experiences (auto) in order to understand cultural experience (ethno)’ (Ellis & Bochner, 2006, p. 273) is paramount – and so an auto-netnographic method was chosen. Inspired by analytical auto-ethnography (Anderson, 2006), the auto-netnography integrated theoretical reflexivity; this enabled a connection of the findings with substantive literature in order to abstract this lived experience and develop new theoretical understandings about the identity of paramedics in primary care. The study design was therefore an analytic auto-netnography, following a similar approach to that outlined by Howard (2018).

#### Investigation

Mapping out the investigative space of the project was inspired by the results of an earlier realist review (Eaton et al., 2021), where searches of Google found posts on the social media platforms of Facebook, Reddit and Twitter (now X) (where the terms featuring ‘paramedics’ and ‘primary care’ were discussed). These posts were ultimately excluded from the realist review, but it was clear that online communities existed on these platforms where paramedics in primary care conversed about their role.

These three data sites offered a range of possible participants to be involved, by virtue of differences between them. Facebook is a social networking service that enables users to connect with others online; Reddit is a website comprising user-generated content in a bulletin board system; and Twitter served as a platform for individuals to connect and communicate by exchanging messages within a restricted character limit. Since April 2023, Twitter has been known as X. This research occurred prior to this date, and so Twitter is referred to herein.

#### Immersion

As a paramedic in primary care, GE shared a common identity, language and experiences with the participants, positioning her as an ‘insider’ within the online participant groups (Asselin, 2003).

Since engagement within these spaces was limited to observation alone, an immersion journal was utilised by GE to respond to posts within these online social spaces and to reflect on these in the context of her own experiences in primary care. This immersion journal employed a first-personal performative writing style (Worden, 2014), within which data was ‘cloaked’ (Kozinets’ term for full anonymisation). This journal served as the primary data source for analysis.

#### Interaction

Given the nature of social media data, interactions focused on observing asynchronous conversations. This lack of immediate interaction allowed the researcher, GE, to adopt a peripheral membership approach, meaning GE was not an active core member but maintained a connection through group membership (Adler & Adler, 1987). This unobtrusive approach was well-suited for understanding the experiences and perceptions of paramedics in UK primary care. It introduced a naturalistic element to the study, enabling GE to join the groups as a fellow paramedic, witnessing and reflecting on participants’ perspectives without influencing the conversations.

#### Integration

The immersion journal was subjected initially to content analysis to generate a broad thematic understanding of the data at a semantic level, following the approach outlined by Braun & Clarke (2022), using NVivo version 12. This analysis was subject to peer debriefing among the authorship team (consisting of clinical academic GPs (GW and KRM) and a health service researcher (ST)).

#### Legal and ethical considerations

Data collection in netnography requires an understanding of social media user rights in terms of compliance with platform policies, data rights and privacy considerations. The study was guided by the underpinning research ethics process flowchart (Kozinets, 2019), with further information outlined in Supplementary 1. This study was approved by the Central University Research Ethics Committee (MS IDREC Ref: R77299/RE001) at the University of Oxford.

## Results

This study was conducted within a strict time frame, taking place between 1 December 2021 and 28 February 2022. During this time, GE spent a cumulative total of 253 hours on social media. The weekly time spent on various social media channels remained consistent, with 7 hours 59 minutes on Facebook, 6 hours 50 minutes on Reddit and 6 hours 42 minutes on Twitter. The additional hour on Facebook could be explained by the author’s participation in more groups on that platform, where much of the chatter between paramedics in primary care was found. GE penned 24,950 words in an immersion journal, which served as the primary source for deriving the main findings. This section provides a narrative overview of the study’s main findings, with italics used to highlight direct quotations from the immersion journal.

### A virtual crew room

An early observation during the engagement with these social media platforms was the general conformity among members across various channels. This phenomenon was notably evident within Facebook groups, which operated like *virtual crew rooms*. These online communities functioned as supportive networks, with members involved in different threads of conversation synchronously, functioning as *a community looking out for each other*. Members used these groups to validate aspects of their clinical practice and seek guidance on integrating their roles into the broader primary care workplace. For the most part, these conversations were characterised by positivity, with responses to queries being supportive and encouraging. A significant portion of the assistance provided revolved around matters such as applying for jobs in primary care, negotiating salaries with practices, structuring working days and hours, development of clinical examination and procedural skills and obtaining support for independent prescribing. The focus was on working together to achieve the best possible conditions for the paramedic to work in within primary care. GE noted that this *dynamic reminded me of the unionised ambulance service workforce that I had encountered in the ambulance station crew rooms during the early part of the last decade (2010‒2019), with a clear ‘us and them’ narrative between the paramedics and their employing practices*.

### Clinical role

When paramedics engaged in conversations about their everyday clinical responsibilities, there was often consistency in how these roles were carried out. This coherence extended to the variety of clinical presentations and skills involved, as well as how they navigated support from GPs in the primary care setting. While the observed spaces generally exhibited coherence, there were instances of direct challenges, primarily related to the justification for individual ability to undertake these clinical roles. These challenges frequently centred around the prescribing rights of paramedics.

### Education

Outside of Health Education England’s ‘Roadmap’ for paramedics in primary care in England (Health Education England, 2021), the lack of standardised UK-wide curriculum caused frustration in discussions. There was a general acceptance that further education is a necessity to work in primary care, but a lack of time within a busy primary care role made it difficult for paramedics to access these opportunities. In this case, enrolment to formal education was viewed as mandatory, but requiring negotiation for completion. In response to a lengthy clinically focused thread that offers an impressive variety of educational resources to support knowledge development among the group:

*Once again, I’m reminded of the importance of this community for sharing resources and building learning. But, for paramedics in primary care not on Facebook, what communities of practice do they belong to? How do they share learning and training opportunities?*

There also existed a fatalistic sense that the role of the paramedic in primary care is not recognised by the wider primary care workforce. Part of this illegitimacy seemed to be around the lack of a standardised curriculum, with frustration that the much-needed standardisation of the education *lands at the feet of policymakers and politicians who have no real-world understanding of what it means to work as a paramedic in primary care*.

### Experience

The absence of formal education led to the majority of knowledge generation happening through hands-on experience, influenced by the hidden work in primary care. Hidden work in primary care refers to non-patient-facing tasks necessary for ensuring patients receive safe and co-ordinated care (Spooner & Hubmann, 2023). Paramedics often found such hidden work, including reporting blood test results and handling clinical administrative tasks, particularly challenging due to the complexities of patient presentations and time constraints:

*My clinical days inevitably ended late. I often could not join the debriefing offered to medical students in the practice whilst I caught up on notes, cleaned or supported colleagues to plan for the following day…*

### Integration into the workforce

It became evident that paramedics in primary care were dedicated to their roles and took pride in being integrated into the primary care team. Stories were shared regarding how employment of paramedics had increased workforce capacity, particularly towards the winter season when demand outstripped available resources. Twitter, in particular, served as a valuable resource where paramedics demonstrated *a keenness and willingness to learn, far in advance of anything I have seen when working in the ambulance service*. It appeared that paramedics were more motivated to grow and assimilate into primary care than they were within the ambulance service culture.

Additionally, there was discourse depicting individuals outside this group of paramedics in primary care (policy makers, clinicians, non-clinicians in primary care and paramedics in the ambulance service) as ‘the other’. This created an ‘us-versus-them’ mentality that appeared to be tacitly acknowledged and accepted in discussions across all social media sites.

### Identity

Some of the discussions made attempts to reconfigure the paramedic professional identity, with members outlining how they no longer felt like a paramedic, as* they bring no unique skills to market in primary care when compared to physiotherapy and nursing. They propose they do the same role as a GP, at a fraction of the cost, with nothing of the same level of experience or training and subsequently doing a worse version of it.*

Alongside this, one post gained traction as the member proposed resigning their paramedic registration to become a physician associate, which they felt more accurately denoted what they did in primary care – assisting the GP, rather than acting as an autonomous healthcare professional. Later, however, in a subsequent comment upvoted by members to replace the first, the respondent outlined that, despite working in the primary care environment, their roots were as a paramedic and that was in their heart too. Responding to this in the immersion journal, GE outlines:

*I really resonate with this; paramedics are not giving up their identity and substituting for other healthcare staff – it’s the origins of paramedics that enable them to work in an advanced practice capacity and be effective at it.*

## Discussion

Analysis revealed that the integration of paramedics in primary care depends on their comfort and perceived competence within their professional role and their ability to align their clinical practice with role requirements through education and workforce integration.

Conversations between paramedics were observed to emphasise the importance of formal education in facilitating integration into primary care. This distinctly Vygotskian perspective indicates that these paramedics view education as a pathway to successful integration into primary care: the more knowledge they acquire, the more patients they can attend to, and the more effective they become in primary care (Vygotsky, 1987).

The exchange of insights concerning clinical practices, daily work routines and support for members seeking guidance with their experiences illustrates how these social media platforms function as tools to assist members in navigating and gaining proficiency in their clinical work in primary care. This social interaction seems fundamental for their cognitive development (Vygotsky, 1987). While predominantly observed in the group-based platforms of Facebook and Reddit, similar posts were also discovered from paramedics on Twitter, showcasing the various methods by which paramedics access and engage within a community of practice using these networks (Lave & Wenger, 1998). In essence, the concept of learning within a community of practice emphasises its foundation in social interactions rather than being restricted to individual cognition (Lave & Wenger, 1998). Considering this within the concept of praxis and experiential learning, it seems that these spaces play a crucial role in the anticipatory socialisation for paramedics entering the primary care workspace (Becker et al., 1977). However, a community of practice should establish standards around which the community develops, such as professional examination or a curriculum.

The lack of formal education in primary care for paramedics poses a risk for those using online platforms. These communities, originally intended for sharing practical experiences, have transformed into practices of communities (Gherardi, 2009), whereby the practical experiences encountered by individual paramedics exchanged on these social platforms have been turned into general principles for *all* paramedics working in primary care. Underpinning this is Goffman’s theory on ‘frame alignment’ (Goffman, 1974), which denotes the process of individuals adjusting their mental frames or cognitive structures to align with others in a social interaction or communication context. Such alignment is crucial for achieving shared understanding and effective communication. However, the use of alignment strategies, which individuals employ to bridge gaps between divergent viewpoints and enhance fluid communication (Goffman, 1974), poses a potential risk of groupthink, characterised as ‘a deterioration of mental efficiency, reality testing, and moral judgment that results from in-group pressures’ (Janis, 1972, p. 9). Janis goes onto say:

*The more amiability and* esprit de corps* among the members of a policy making in-group, the greater is the danger that independent critical thinking will be replaced by groupthink, which is likely to result in irrational and dehumanizing actions directed against out-groups.* (Janis, 1972, p. 13)

The concept of ‘othering’ was evident in some conversations observed for the study, where paramedics distanced themselves from other members of the primary care team and paramedics in ambulance services, creating a situation where ‘other’ paramedics are perceived as a separate and potentially inferior profession. Considered within a community-of-practice framework, it is conceivable that paramedics in primary care define their identity through the distinct environment in which they work.

The paradoxical observation emerges that paramedics in primary care perceive their identity as notably different from their ambulance counterparts, leading to a sense of ‘othering’, while still retaining the fundamental characteristics of paramedicine. It is possible that paramedics will reconcile the internal dualism of thrill-seeking traits with those of caregiver (Donnelly et al., 2015) as they seek to harmonise their ‘blue-collar’ trade with autonomous professional in primary care (McCann, 2022). Nevertheless, this reconciliation does little to produce a stable professional identity. The pursuit of professional growth and expanding autonomy in a setting not commonplace with the paramedic profession carries the potential to trigger an identity crisis, akin to a state of Kierkegaardian existential uncertainty (Hill & Eaton, 2023; Kierkegaard, 1843). When paramedics move from a ‘blue-collar’ ambulance-based role into a professional workspace, such as primary care, this creates tension in the expectations paramedics have about their role and how that role should be practised (Granter et al., 2018). Role identity arises from an individual’s engagement and integration within their environment (such as primary care), as well as their community of practice (such as these online spaces) (Stryker, 1980). Therefore, the formation of role identity requires that an equilibrium exists between social structures and individual agency (Archer, 1995). This resonates with the notion that paramedics are actively redefining their professional identity while working in primary care to integrate with the context of this work environment. Role dissonance emerges when there is a conflict between the envisioned concept of work and the actual execution of work (Hollnagel et al., 2015). This disparity arises from the insufficient preparation of paramedics for the practical intricacies of primary care, further exacerbated by the absence of a formal education pathway. It is plausible that paramedics who articulated their identity in primary care within these online social spaces have managed to reconcile this dissonance, fostering an understanding of their role within this workspace (Mausz et al., 2022).

### Strengths and limitations

Auto-netnography represents a highly personalised exploration for individuals to scrutinise their practice, existence and role within the context of their study (Kozinets & Kedzior, 2009). Through observational data collection within a reflexive immersion journal, GE gained insights into both herself and her role as a paramedic in primary care that go beyond what she could have discovered solely through introspection or observation of others alone – a point supported in other work (Dwyer & Buckle, 2009; Sidebotham, 2003; Yeo & Dopson, 2018).

It is crucial to note that just because members were part of online communities for paramedics in primary care, it does not guarantee that they were paramedics working in primary care. Similarly, this study only captures the perspectives of paramedics in primary care who actively engage on social media platforms. It is important to acknowledge that many paramedics working in primary care may not use social media or participate in online forums and, as a result, their perspectives and experiences are not represented in this type of research. However, since the opinions expressed across these data sites aligned with GE’s own experiences as a paramedic in primary care, it is likely that members participating in these forums are generally representative of paramedics in primary care.

Finally, the observation of online social spaces occurred asynchronously, taking place after the conversation had concluded, unlike synchronous participation as an active contributor in the discourse. The value of the insights acquired is inherently linked to the quality of the interactions witnessed within these spaces. Consequently, this aspect can pose difficulties when attempting to extend findings to groups beyond those directly observed. Nevertheless, by using three distinct data sites, each with its unique operational characteristics and thus catering to a diverse array of individuals, it is anticipated that this limitation has been effectively addressed.

## Conclusions

Utilising analytic auto-netnography proved essential for the practitioner-researcher to transcend her personal experiences, allowing exploration into the perceptions of other paramedics in primary care regarding their work and professional identity. Adopting a peripheral membership approach in engaging with paramedics on three social media forums facilitated a reflexive stance, rather than an active one.

Findings from this research outline that, in the absence of formal education, paramedics learn through a combination of praxis, clinical supervision and an online community-of-practice network facilitated by other paramedics in primary care. Within such a community, issues of the professional identity of paramedics working in primary care, and their place within the profession, are noted.

## Acknowledgements

Georgette Eaton would like to thank Associate Professor Kathryn Eccles, Oxford Internet Institute, for initial support in understanding the ethical components underpinning this methodology; Professor Julia Williams, University of Hertfordshire, for intellectual guidance in developing the protocol; and Veronika Williams, Nipissing University, for intellectual guidance in developing the protocol and applying for ethical approval.

## Author contributions

GE incepted the study and led on developing the protocol, applying for ethical approval, all stages of data collection and management and analysis, and led on writing the article. GW was involved in developing the protocol, applying for ethical approval, decisions on data analysis and writing the article. ST was involved in developing the protocol, applying for ethical approval, decisions on data analysis and writing the article. KRM was involved in developing the protocol, applying for ethical approval and writing the article. All authors read and approved the final manuscript. GE acts as the guarantor for this article.

## Conflict of interest

None declared.

## Ethics

Approval for this study was obtained from the Central University Research Ethics Committee (MS IDREC Ref: R77299/RE001) at the University of Oxford.

## Funding

GE is supported by a National Institute for Health Research (NIHR) Doctoral Research Fellowship (NIHR300681).
